# Optimization of Reinforcing Patch Effects on Cracked Plates Using Analytical Modeling and Taguchi Design

**DOI:** 10.3390/ma16124348

**Published:** 2023-06-13

**Authors:** Abdul Aabid

**Affiliations:** Department of Engineering Management, College of Engineering, Prince Sultan University, P.O. Box 66833, Riyadh 11586, Saudi Arabia; aaabid@psu.edu.sa

**Keywords:** damaged plate, reinforcing patch, stress intensity factor, design of experiments, analytical modeling

## Abstract

Over the past four decades, the use of composite materials for the repair of cracked structural plates with glued patches has been extensively studied. Attention has been focused on determining a mode-I crack opening displacement, which is important in tension load and in preventing the failure of a structure due to small damages. Therefore, the significance of conducting this work is to determine the mode-I crack displacement of the stress intensity factor (SIF) using analytical modeling and an optimization method. In this study, an analytical solution was obtained for an edge crack on a rectangular aluminum plate with single- and double-sided quasi-isotropic reinforcing patches, using linear elastic fracture mechanics and Rose’s analytical approach. Additionally, an optimization technique with the Taguchi design was used to define the optimal solution of the SIF from the suitable parameters and levels. As a result, a parametric study was conducted to assess the mitigation of the SIF using analytical modeling, and the same data were used to optimize the results via the Taguchi design. This study successfully determined and optimized the SIF, demonstrating an energy- and cost-efficient approach to address damage control in structures.

## 1. Introduction 

Aluminum alloys are the most predominantly used materials and have been used in early studies as aircraft structural materials [1]. However, composite materials have a significant impact on the application of aerospace structures because of their efficient properties for manufacturing [2] and damaged aircraft structural repair purposes. Therefore, passive repair methods have been widely used over the last four decades for unhealthy/damaged structures [3], and they have also been reviewed by the experts in this research considering their significance and limitations [4,5]. Hence, the impact of a composite patch on an aluminum cracked plate was investigated using different approaches such as analytical [6,7], finite element [8,9], experimental [10,11], and Taguchi design [12,13,14] methods. These studies showed that center, edge, patched, and unpatched plate models produced SIF reduction [15]. In some cases, using a p-convergent layered modeled repair was performed on the damaged plate, which showed that the composite patch had an approximately 50% reduction in the SIF [16]. Bouiadjra et al. [17] demonstrated the performance of a double-sided composite patch on a damaged plate. According to their investigations, a single-sided patch is more efficient than a double-sided patch, and the amount of reduction has been proven to be nearly 30%.

Considering the material property and loading conditions, Rabinovitch and Frostig [18] explored a variety of composite patches that are accessible. Compression stresses in bonded strips tend to cause buckles, start cracks, and accelerate the cracks’ unstable propagation into the unbonded area of the split plate. Therefore, understanding the patch size is crucial, as a high patch thickness would cause a significant reduction in the SIF, yet a higher thickness would increase the weight. Consequently, the patch and adhesive thickness should be kept to a minimum. The most commonly utilized composite materials for the restoration of fractured material are the glass-epoxy, graphite-epoxy, and boron-epoxy compositions [19]. Albedah et al. [20] analyzed the effects of single- and double-sided circular shapes of patches for the repair of damaged structures and compared their performance using the FE method. The SIF under the temperature effect for a patched aluminum plate with a glued composite patch was measured by Ergun et al. [21] using a combination of evolutionary algorithms and FEM. In addition, Kwon et al. [22] employed a composite patch to mend a broken plate on one side and reduce the strain energy release rate (SERR). They established an analytical model for calculating the SERR of a damaged base plate.

In recent investigations on the bonded composite repair of damaged structures, numerous studies have reported using the FE method [23,24,25] and experimental work [26] compared with analytical work. Additionally, the stress concentration factor (SCF) has played an important role in fracture parameters, and it has been reduced using a bonded composite patch with the same FE method [27,28]. To reduce the SIF, researchers have also utilized different bonded material combinations such as piezoelectric and composite materials together [29]. Regarding bonded composite repair, Baker [30] examined the crucial aspects of certifying such repairs in situations where the patch is responsible for restoring the residual strength of flight-critical structures. To evaluate the durability and damage tolerance of bonded joints, Baker listed stress values under varying conditions and compared them with those of the lower wing made of the aluminum alloy 2024 T581.

In some investigations, the importance of reinforcing patch materials was observed by varying the shapes that were applied under various loading conditions [31,32]. Another study investigated an internally stiffened foam-filled reinforced composite under impact loading for energy absorption applications [33]. The current review is conducted based on the host and reinforcing materials and structures and the used methodologies. After conducting a literature review, several studies have been found on reducing the SIF using different techniques and approaches. Moreover, an analytical approach was found for single-sided composite repair, but to the author’s best knowledge, no studies have been shown in the literature in which double-sided reinforcing patch repair has been performed using LEFM and Rose’s analytical approach and optimized using the Taguchi design method. Hence, to fill this gap, the present study focused on a novel analytical approach for the double-sided composite repair of an edge-cracked plate and compared it with a single-sided reinforcing patch. Additionally, optimization studies were presented to examine the mitigation of the SIF and compared with existing results, resulting in an energy-saving and cost-efficient approach.

## 2. Problem Formulation

**Figure 1:**
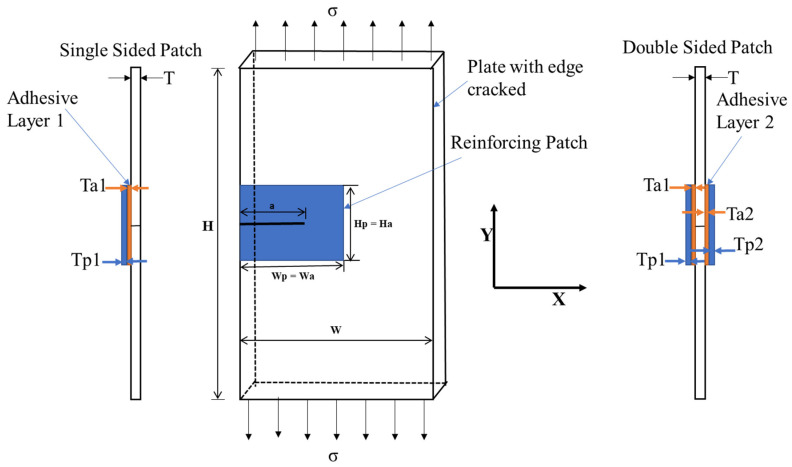
Rectangular plate with an edge crack integrated with a reinforcing patch.

This study focused on a thin aluminum plate with a uniform edge crack under plane stress conditions, as depicted in Figure 1. The dimensions of the edge-cracked rectangular plate were 200 mm in height (H), 40 mm in width (W), and 1 mm in thickness (T). The default value for the crack length (a) in the current model was set to 10 mm, with an applied tensile stress of 1 MPa. Both the adhesive bond and reinforcing patch had a height of 20 mm (Ha = Hp) and width of 20 mm (Wa = Wp), with a thickness of 0.03 mm (Ta1 = Ta2) and 0.5 mm (Tpa1 = Tp2) on both sides, respectively. To address the crack, a reinforcing patch was applied to the crack field, with dimensions that were nearly twice the size of the crack area to effectively close the crack length. The mechanical properties of the edge-cracked rectangular plate A2024-T3 and adhesive layer included density values of 2715 kg/m^3^ and 1160 kg/m^3^, Poisson’s ratios of 0.33 and 0.3, and Young’s moduli of 68.95 GPa and 5.1 GPa. 

The elastic properties of a quasi-isotropic reinforcing patch under generalized plane stress can be specified with four constants, which are usually taken to be the principal Young’s moduli $E_{x}$ and *E_y_* the major Poisson’s ratio v (=$v_{xy}$), and the shear modulus G (=$G_{xy}$). There are two Poisson’s ratios, $v_{xy}$ and $v_{yx}$, which are related via $v_{yx}E_{x}=E_{y}v_{xy}$ [6,34]. We chose $v_{xy}$ as the major Poisson’s ratio, having in mind applications in which the “fibre direction” is parallel to the *x*-axis (then, $E_{x}$ > $E_{y}$). An isotropic plate can be considered a special case of an orthotropic plate for which E = $E_{x}$ = $E_{y}$, $v_{xy}=v_{yx}=v$, and the shear modulus is no longer an independent constant but is related to Ev via E=2G(1+v). Furthermore, the Y moduli of the plate and the reinforcement were both assumed to be isotropic and to have almost the same Poisson’s ratio. The mechanical properties of the reinforcing patch included a density value of 2000 kg/m^3^, Poisson’s ratio of 0.33, and Young’s modulus of 200 GPa.

The shear modulus of the adhesive bond was 1.2 GPa, and that of the reinforcing patch was 7.5 GPa. The composite was defined as a quasi-isotropic patch, and the relevant parameters of the patched system were chosen to determine the SIF in the repaired plate [6,35]. The choice of composite material was based on its lightweight nature and high resistance ratio, leading to the selection of this composite for this investigation. This material exhibits excellent characteristics in shear stress transfer, as reported in previous studies [36,37,38].

While a rectangular shape for a patch is not considered the most optimal option by Mahadesh and Hakeem [39], the current study considered mechanical effectiveness and development-related factors. The patch size was the result of numerous numerical experiments, which are not discussed in detail herein for the sake of brevity. However, the selected patch size effectively covered the length of the crack and improved mechanical behavior under loading situations [35]. We considered some limitations to solve this problem: In particular, the adhesive stayed elastic, and any plastic deformation at the fracture tip was limited to a tiny zone (compared with the crack length); thus, it could be disregarded when calculating the SIF. All deformations were linearly elastic. It was possible to imagine the elastic condition of the reinforcement and the plate as states of generalized plane stress, disregarding any change in the thickness. According to the classical theory of bonded joints, the adhesive layer acted as a shear spring (see Section 3.2).

## 3. Mathematical Modeling

### 3.1. Stress Intensity Factor for Cracked Plate without Reinforcing Patch

In the fracture mechanics studies, the expression to calculate the stress intensity factor with the linear elastic fracture mechanics method for an infinite cracked plate was identified, where mode-I (the opening) was deemed the only useful mode under uniaxial tensile load. A linear elastic material’s stress distribution at the crack’s tip is characterized using the SIF. To identify the SIF for a finite plate with any crack length, the polynomial expression to fit the solution was determined via the geometrical factors F(a/W) for the plate. Due to the finite structure of the plate, the boundary condition places more stress near the crack’s tip. The edge-cracked plate’s mode-I SIF is provided via [40]

(1) $$K_{I}=\sigma_{0}\sqrt{\pi a}\;\sqrt{\left[\frac{2W}{\pi a}\tan\frac{2a}{2W}\right]}\frac{0.752+2.02\left(\frac{a}{W}\right)+0.37{\left(1-\sin\frac{2a}{2W}\right)}^{3}}{\cos\frac{2a}{2W}}$$

where

(2) $$F\left(\frac{a}{w}\right)=\sqrt{\left[\frac{2W}{\pi a}\tan\frac{2a}{2W}\right]}\frac{0.752+2.02\left(\frac{a}{W}\right)+0.37{\left(1-\sin\frac{2a}{2W}\right)}^{3}}{\cos\frac{2a}{2W}}$$

where $\sigma_{0}$ is the uniaxial uniform tensile load, which is perpendicular to the length of the crack ‘a’.

### 3.2. Stress Intensity Factor for Cracked Plates with Single and Double Reinforcing Patches

The passive strengthening impact due to a patch glued to a damaged plate under uniform tensile stress, $\sigma_{0}$, can be equivalent to the reduced uniform tension stress, $\sigma_{p}$, acting on the bonded surface of the edge-cracked plate through the superposition principle [41,42]. The uniform uniaxial tensile stress is equal to the normal stress along the potential crack path in the bonded reinforcement zone of the composite-reinforced healthy aluminum plate. The uniform tension stress must be determined to evaluate the SIF reduction. 

For an edge-cracked plate under uniform tensile stress ($\sigma_{0}$) and the additional force shared between the plate, bond, and patch, two assumptions were made [42] to identify the reduced uniform tension stress ($\sigma_{p}$):

1. The adhesive bond’s shared tension stress was very negligible and overlooked.
2. The plate and patch both experienced the same tension strain since there were no force-lag effects between the two materials.

**Figure 2:**
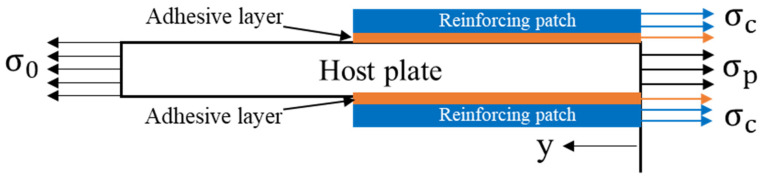
The stress component of the infinite uncracked aluminum plate was reinforced with a reinforcing patch.

The reinforced uncracked plate’s stress components are shown in Figure 2, and it is possible to obtain the following expression from the force equilibrium equations:

(3) $$\sigma_{0}A_{p}=\sigma_{p}A_{p}+2\sigma_{c}A_{c}$$

where $A_{p}$ is the cross-sectional area of the rectangular plate; $A_{c}$ is the cross-sectional area of the patch; and $\sigma_{c}$ is the tensile stress of the patch. Since the two plates had two sides, they were multiplied by two in Equation (3).

The reinforcing patch bonded on (above) the plate crack length that closes the damaged area at both sides and the SIF can be expressed for a plate under uniaxial tensile load as

(4) $$K_{I}=\sigma_{p}\sqrt{\pi a}$$

In a cross-section configuration, it was presumed that the allocation of stress in the uncracked plate was approximately the same. The one-dimensional bonded joint theory [43] was used explicitly to obtain the stress $\sigma_{p}$. For double-sided repair with reinforcing patches, the reduced stress is expressed as

(5) $$\sigma_{p}=\frac{\sigma_{0}}{1+S},$$

where S represents the stiffness ratio between the bonded patch and cracked plate, and it is written for single-sided patch [44] as

(6a) $$S=\frac{E_{c}^{\prime }t_{c}}{E_{p}^{\prime }t_{p}},$$

where $t_{p}$ represents the plate thickness, $E_{p}^{\prime }\;$represents the modulus of elasticity of the plate, $E_{c}^{\prime }\;$represents the modulus of elasticity of the patch, and $t_{c}$ represents the patch thickness. For a double-sided patch, it was assumed that the plate stiffness was directly proportional to twice the patch stiffness considering both sides/phases of the plate. Therefore, it was written in the form of

(6b) $$S=\frac{2E_{c}^{\prime }t_{c}}{E_{p}^{\prime }t_{p}},$$

Generally, Equation (6b) was multiplied by two times the patch stiffness since the plate has both sides of the patch. However, no studies have been presented in the literature that provide an analytical solution for a double-sided patch. To validate the obtained results from Equation (6b), a comparison was made with existing experimental and FEM results. This was carried out to demonstrate the accuracy and reliability of the current model, where Young’s moduli for plane strain and plane stress conditions are

$$\mathrm{Plane}\;\mathrm{strain}:\;E_{c}^{\prime }{\;\mathrm{or}\;E}_{p}^{\prime }=\frac{E_{c}{\;\mathrm{or}\;E}_{p}}{\left(1-{\;v}^{2}\right)},\;\mathrm{and}\;\mathrm{plane}\;\mathrm{stress}:\;E_{c}^{\prime }=E_{c}\;\mathrm{or}\;E_{p},$$

It was observed that the tensile stress in the reinforced zones could be reduced with bonded reinforcing patches, and the reduction mainly depended on the stiffness ratio. 

**Figure 3:**
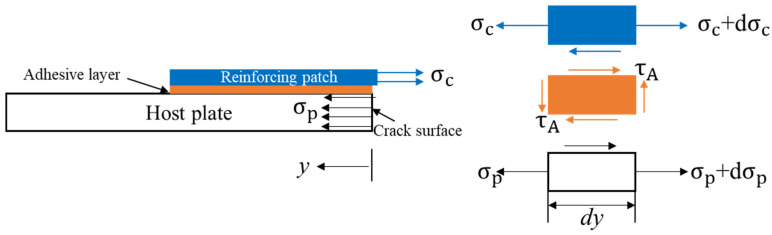
Stress analysis of the infinite edge-cracked aluminum plate reinforced with a reinforcing patch.

Considering the symmetry requirement of the plate quarter model depicted in Figure 3, to make the solution easier, two suppositions were employed [42]:

1. Only shear deformation of the adhesive bond was observed, and the adhesive thickness and shear stress were distributed equally.
2. Flexural deformation of the plate and the patch was ignored in preference for the elastic continuum represented by the plate and patch.

The equilibrium equation of the reinforcing patch and the aluminum plate can be written as

(7) $$\frac{{d\sigma }_{c}}{\mathrm{dy}}-\frac{\tau_{A}}{t_{c}}=0$$

(8) $$\frac{{d\sigma }_{p}}{\mathrm{dy}}+\frac{\tau_{A}}{t_{p}}=0$$

where $\tau_{A}$ represents the shear stress of the adhesive bond. Since the plate thickness $t_{p}$ was thin, it was assumed that the present work was under a plane stress state, and the stress–displacement relationship of the patch and the plate were

(9) $$\sigma_{c}=E_{c}\varepsilon_{c}=E_{c}\frac{{\mathrm{du}}_{c}}{\mathrm{dy}},$$

(10) $$\sigma_{p}=E_{p}\varepsilon_{p}=E_{p}\frac{{\mathrm{du}}_{p}}{\mathrm{dy}},$$

respectively, where $u_{c}$ represents the longitudinal displacement of the patch, $u_{p}$ represents the longitudinal displacement of the plate along the *y*-axis, and $\varepsilon_{p}$ and $\varepsilon_{c}$ are the strain of plate and patch, respectively. The substitution of Equations (9) and (10) into Equations (7) and (8) can be solved as

(11) $$\frac{d^{2}u_{c}}{{\mathrm{dy}}^{2}}-\frac{d^{2}u_{p}}{{\mathrm{dy}}^{2}}=\tau_{A}\left(\frac{1}{E_{p}t_{p}}+\frac{1}{E_{c}t_{c}}\right)$$

According to the deformation compatibility between the plate, bond, and patch, the following equation can be obtained [42]:

(12) $$\gamma_{A}=\frac{u_{c}-u_{p}}{t_{A}}$$

where γ_A_ represents the shear strain, and $t_{A}$ represents the thickness of the adhesive bond. The relation between shear stress and shear strain can be expressed as

(13) $$\tau_{A}=\gamma_{A}G_{A}=G_{A}\frac{u_{c}-u_{p}}{t_{A}},$$

where $G_{A}$ represents the shear modulus of the adhesive bond. Combining Equations (11) and (13), the governing differential equation for the shear stress in the adhesive bond can be derived as

(14) $$\frac{d^{2}\tau_{A}}{{\mathrm{dy}}^{2}}-\beta^{2}\tau_{A}=0,$$

where β represents the shear stress transfer length in a delegate bonded joint and is given via

(15) $$\beta =\sqrt{\frac{G_{A}}{t_{A}}\left(\frac{1}{E_{p}t_{p}}+\frac{1}{E_{c}t_{c}}\right)},$$

By solving Equation (15), the expression for the shear stress is obtained [41]:

(16) $$\tau_{A}=\tau_{A}^{\max}e^{-\beta y},$$

where $\tau_{A}^{\max}$ represents the maximum shear stress at the end of the adhesive bond.

With a reinforcing patch, the length of a crack is fully bonded on one side in an infinite plate; hence, the distribution of the shear stress will occur in the y-direction, and Rice [45] expressed the upper bond SIF for an infinite cracked plate with the physical parameter ‘c’, and the expression is

(17) $$K_{I}=\sigma_{p}\sqrt{\pi c},$$

where

(18) $$c=\frac{1}{\pi k},$$

and k represents the spring constant and is given via

(19) $$k=\frac{\beta S}{\left(1+S\right)\left(1-v^{2}\right)},$$

The substitution of Equation (20) into Equation (19) can be written as

(20) $$c=\frac{\left(1+S\right)}{S}\cdot \frac{\left(1-v^{2}\right)}{\pi \beta }$$

where ‘v’ represents the Poisson’s ratio of the cracked plate. Equation (17) is derived for an infinite length of a crack, so the obtained SIF is for the upper bond to the infinite cracked plate.

Due to the reinforcement effect of the patch, these approximations are conservative. Hence, the calculation results can be regarded as the lowest SIF bound for an infinite cracked plate reinforced with a single-sided patch [46]. To calculate the SIF for an infinite cracked plate using a single-sided patch, the following expression was created for an arbitrary crack length [43,46]:

(21) $$K_{I}=\sigma_{p}\sqrt{\frac{\pi ac}{a+c}}=\sqrt{\frac{c}{a+c}}\cdot \sigma_{p}\sqrt{\pi a},$$

It can be observed that the results obtained from Equation (21) reach the results from Equation (1) when the crack is very short. When the crack length is very long, the estimated values of Equation (21) reach the results from Equation (17). This study confirms that Equation (20) is within the upper and lower limits. 

By substituting Equation (5) into Equation (21), the SIF with the crack length under uniaxial tensile stress for the cracked plate reinforced with a patch can be easily determined with the following equation:

(22) $$K_{I}=\frac{1}{1+S}\sqrt{\frac{c}{a+c}}\cdot \sigma_{0}\sqrt{\pi a}=\delta_{1}\delta_{2}\sigma_{0}\sqrt{\pi a},$$

where

$$\delta_{1}=\frac{1}{1+S}\;;\;\delta_{2}=\sqrt{\frac{c}{a+c}}$$

In the SIF solution, two correction factors, i.e., $\delta_{1}$ and $\delta_{2}$, are used in comparison with the SIF solution for the infinite cracked aluminum plate that was added to account for the composite reinforcement effect. The values of both correction factors were less than one, indicating that the edge-cracked plate to be taken into consideration for the patch might lower the SIFs. The main source of the reinforcing effect of reinforcing patches is the reduction in tension and restriction of the crack opening [41,42]. The uniform uniaxial tensile stress reduction effect, which is mostly dependent on the stiffness ratio ‘S’, is represented by the first correction factor ‘$\delta_{1}$’. Smaller SIF values can be produced via a higher stiffness ratio ‘S’. The crack’s constraint effect is shown via the second correction factor $\delta_{2}$, which mostly depends on the crack’s length, stiffness ratio, shear modulus, and bond thickness. By raising the stiffness ratio and the adhesive shear modulus and/or decreasing the adhesive thickness, the SIF can be reduced.

For an infinite plate with the vital crack problem in linear elastic fracture mechanics (LEFM), the solution is obtained using Equation (22). The geometry dimensional function F(a/w) becomes significant when the crack body geometry has an impact on the crack length. Using the empirical formula proposed by Tada et al. [40] for a finite edge-cracked rectangular plate, the equation is thus rewritten in the form of

(23) $$K_{I}=\delta_{1}\delta_{2}\sigma_{p}\sqrt{\pi a}\sqrt{\left[\frac{2W}{\pi a}\tan\frac{2a}{2W}\right]}\frac{0.752+2.02\left(\frac{a}{W}\right)+0.37{\left(1-\sin\frac{2a}{2W}\right)}^{3}}{\cos\frac{2a}{2W}}$$

## 4. Taguchi Design

In the early stages of research, the Taguchi design was applied in the manufacturing industry to identify suitable factors for the production process [47,48,49]. From the planning of industrial products to conducting experiments, the Taguchi design plays a significant role in various industries. It helps to reduce human effort and guide the selection of significant factors and their levels for the designer. In the Taguchi design, a variety of components that the designer can control and that can be changed at two or more levels are effectively utilized. The optimization method provides an optimal design for the experiments, whereby each significant parameter combination is tested for the potential response factor. Over the last few decades, several studies have shown the effectiveness of the Taguchi design approach, particularly in civil and mechanical engineering, in optimizing data. Hence, in this work, we utilized the Taguchi design method to optimize the repair model parameters for the best SIF response. 

### Taguchi’s Orthogonal Array

To address issues in experimental design, Taguchi proposed highly fractionated factorial designs with additional orthogonal arrays (OAs) and some innovative arithmetic techniques. This resulted in a series of results and discussions, but the fact that Taguchi’s methodology was primarily promoted by engineers and lacked sufficient peer review contributed to some issues. In the late 1980s, peer review findings showed that while Taguchi’s engineering goals and concepts were well-founded, his experimental design and data analysis techniques had serious flaws.

For the current type of tests, a robust design is typically well-ordered. Based on the Taguchi design, the design can identify every combination of components that can be used in a single or repeated trial of work. The major impact refers to a difference in response caused by a change in the level of elements, which is a crucial aspect of the interest in such work.

**Figure 4:**
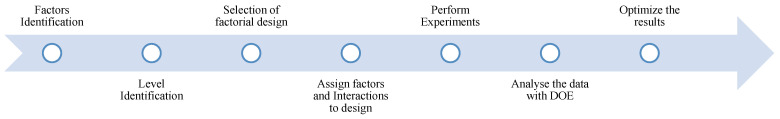
Steps in implementation of the Taguchi design approach.

Assuming a linear problem for this investigation, Taguchi’s OA was planned and set up with 4 selected parameters and 2 levels (P = 4 and L = 2), as shown in Table 1. Figure 4 illustrates the overall Taguchi design approach procedure, and Table 1 shows a well-organized typical OA with all possible combinations. In this work, design factors such as patch type, crack length, patch thickness, and applied load of the repair model were chosen, and the fracture parameter SIF was considered the response parameter. Table 2 shows that a total of eight runs of analytical cases were performed based on the chosen factors and their levels. For the Taguchi OA, the first column was set to the patch type with a categorical factor, while the crack length, patch thickness, and applied load were considered numeric factors, as shown in Table 3. After performing the analysis, the SIF of each input was added to Table 4.

This would be a determination of all the impacts, such as first interactions when running a Taguchi OA. However, the current study focuses on the main findings from the ANOVA and regression analyses, and the linear model is as follows.

(24) $$Y=b_{0}+b_{1}X_{1}+b_{2}X_{2}+b_{3}X_{3}$$

where

Y= the dependent variable (dimensional SIF);

$b_{0}=$ the intercepts or reaction variables of the SIF at the crack’s tip; 

$b_{1},{\;b}_{2},{\;\mathrm{and}\;b}_{3}=$ the linear regression coefficients of the patch type, crack length, patch thickness, and applied load.

Taguchi orthogonal arrays (OAs) are recommended over a full factorial array analysis, as the latter would be expensive and time-consuming, with less emphasis on precision. An OA is typically used in industrial applications to investigate the impacts of various control parameters. Additionally, the columns for the independent variables are orthogonal to one another in this type of study. When describing an OA, several layers and factors must be considered. In this study, four parameters in each group had their degrees of freedom (DF) calculated at various values of one. Therefore, eight runs of a two-level Taguchi OA were chosen. The experiment’s total DF was 8 − 1 = 7.

## 5. Results and Discussion

### 5.1. Benchmark Validation of the Present Analytical Model

To validate the accuracy of a mathematical model for single-sided repair, existing benchmark results were replicated using a similar model and parameters [11]. The test specimen was manufactured by Lexas (PCBA), with an edge crack, and the bonded patch was made from Plexiglas (PMMA), under a uniform tensile load. The dimensions of the cracked plate were 0.100 m in width, 0.200 m in length, and a thickness of 0.002 m, with a damaged length of 0.040 m. Similarly, the bonded patch had a width of 0.100 m, and the length and thickness were considered in two ranges, 0.080 m and 0.200 m, and 0.002 m and 0.004 m, respectively.

**Figure 5:**
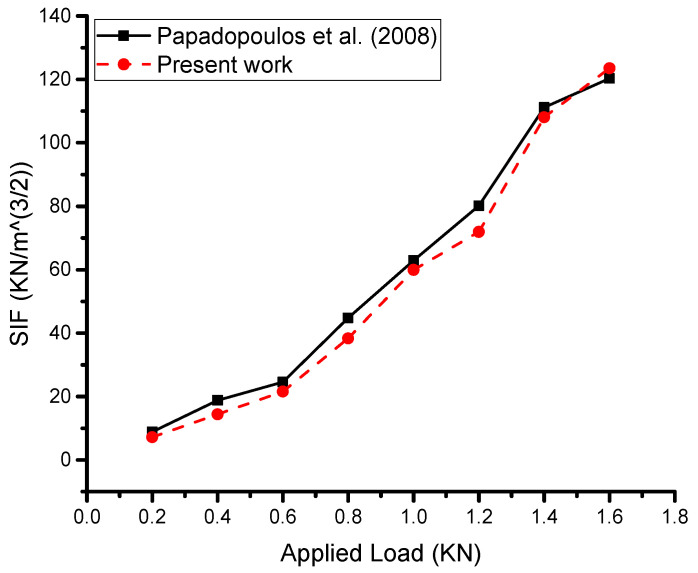
Validation of the current analytical model (single-sided reinforcing patch) [11].

The current work involved analytical modeling and analysis, which demonstrated very good results compared with the experimental work. Proper modeling and a theoretical background were crucial in achieving these results, while the boundary conditions were the most critical part of the analytical work. The present work (Figure 5) showed that the mathematical model performed very well, with a relative error of less than 10% between the current work and the experimental work of Papadopoulos et al. [11]. This suggests that our results are reasonably robust and that the input data used in the analysis are consistent with the experimental data.

The geometry of the cracked structure analyzed by Belhouari et al. [9] shares similarities with the one depicted in Figure 1, featuring a central crack length (a) of 25 mm and subjected to a remote uniaxial tensile load of 70 MPa under plane stress conditions. We used the same dimensions and properties of the problem as Belhouari et al. [9]. The basic geometry of the cracked structure considered in this study is shown in Figure 1. An aluminum plate with a height of 254 mm, width of 127 mm, and thickness of 5 mm was repaired with a boron–epoxy composite with a height of 75 mm, width of 65 mm, and thickness of 5 mm, and the adhesive bond FM 73 with a height of 75 mm, width of 65 mm, and thickness of 0.15 mm was used to bond the plate and patch. The material properties of the plate included Young’s modulus of 72 GPa and Poisson’s ratio of 0.33. The patch Young’s modulus was Er_1_ = 210 GPa, Er_2_ = Er_3_ = 19.6 GPa, and the Poisson’s ratio was ʋr_1_ = 0.3, ʋr_2_ = ʋr_3_ = 0.2, and the shear modulus was 5.460 GPa. The adhesive bond had a shear modulus of 0.42 GPa.

To conduct the analysis, Belhouari et al. [9] used the Franc2D/L code developed by Kansas University [50], which employs finite-element configurations, as shown in Figure 1.

**Figure 6:**
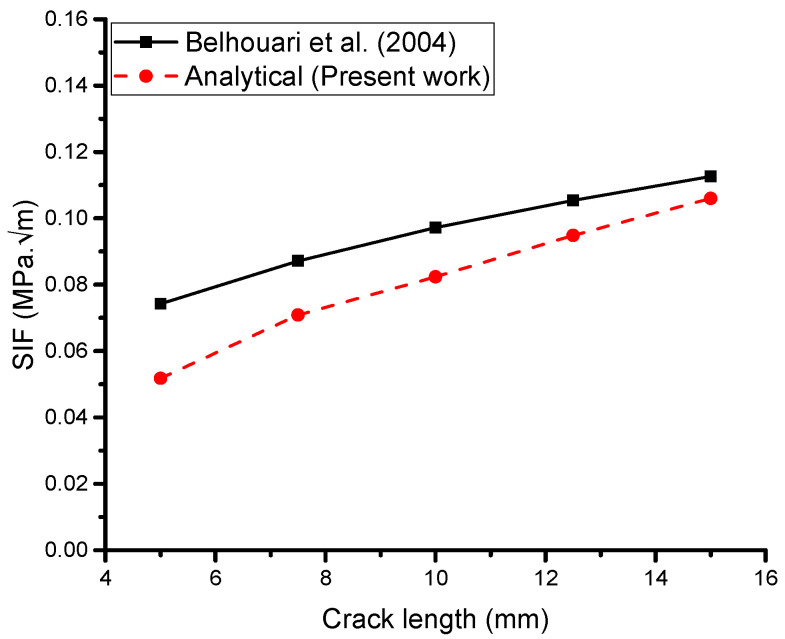
Validation of the current analytical model (double-sided reinforcing patch) [9].

In any analytical or numerical analysis, it is important to use accurate and consistent input data such as material properties, boundary conditions, and loading conditions. The consistency of the input data with the experimental data is critical to ensure the validity and reliability of the analysis results (Figure 6).

### 5.2. Reduction in and Comparison of SIF Performance

To investigate the performance of the SIF using the mathematical formulation for a single- and double-sided patch, three key parameters were considered. 

#### 5.2.1. Effect of Crack Length

**Figure 7:**
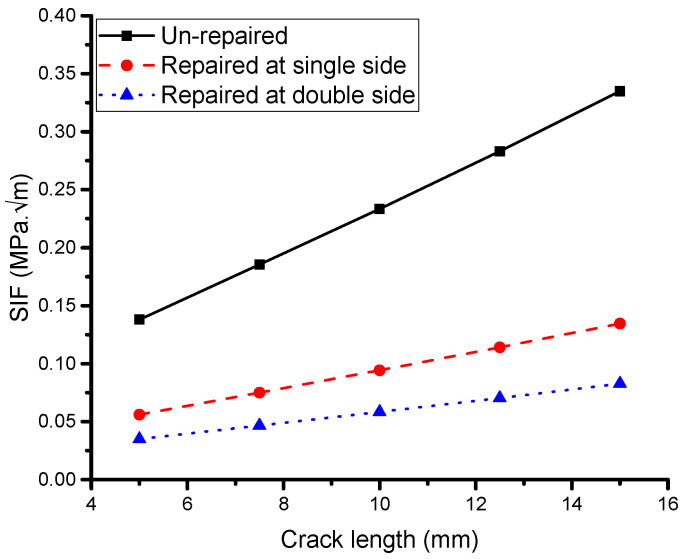
Effect of crack length.

Figure 7 depicts the variation in the SIF for single and symmetric double-sided patches with a thickness of 0.5 mm as a function of the crack length. Firstly, it is evident that both cases exhibit asymptotic behavior, and the use of a double-sided symmetric patch reduces the asymptotic value by approximately 10%, suggesting that the fatigue life of the structure can be significantly enhanced. This trend is consistent with the results reported by Klug et al. [51] and Belhouari et al. [9], who found that using a double-sided patch can extend the fatigue life of a structure twice as much as using a single-sided patch. Additionally, as shown in Figure 7, there is no change in the SIF between the single and double symmetric patch cases for low values of crack length. Belhouari et al. [9] investigated crack lengths ranging from 0 to 5 mm and found no significant effect on the reduction in the SIF. This is because at low thickness values, the stress intensity at the crack’s tip is insufficient to emphasize the advantage of the double stress transfer. However, a reduction in the SIF is observed compared with the unrepaired plate, and for higher crack lengths, a higher reduction in the SIF is achieved via both single- and double-sided composite repairs, although the effect is less pronounced in the case of double-sided repairs than in the case of single-sided repairs. 

#### 5.2.2. Effect of Patch Thickness

**Figure 8:**
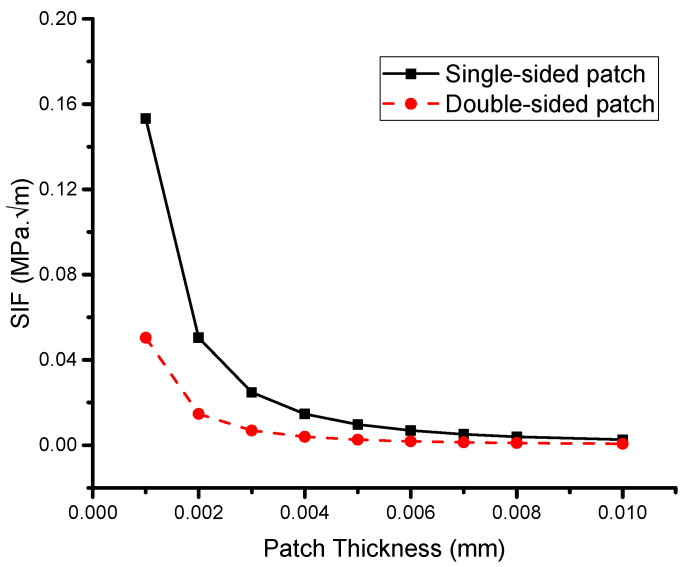
Effect of the patch thickness.

**Figure 9:**
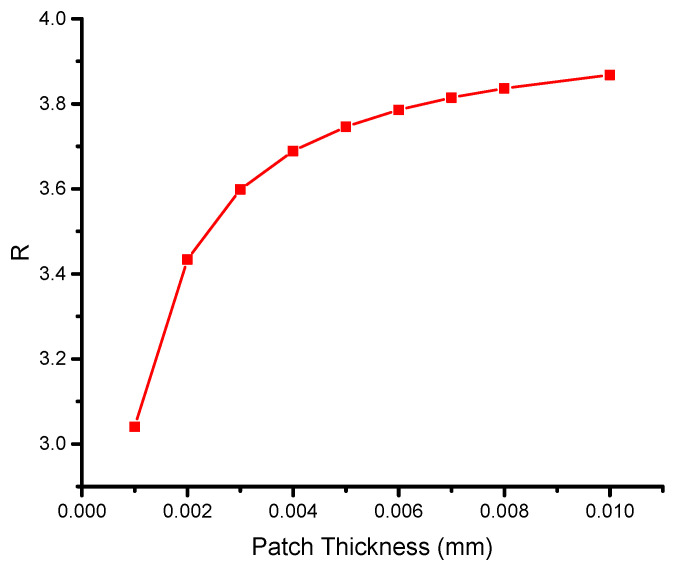
Variations in the ratio R according to the patch thickness.

Figure 8 illustrates the variation in the asymptotic SIF with patch thickness for single and double patches, highlighting the superior performance of the double-sided reinforcing patch. As the patch thickness increases, the SIF in both cases drops asymptotically. Notably, the maximum thickness of a single- or double-sided reinforcing patch leads to a similar value, and further increases in patch thickness do not yield any additional reduction in the SIF. Therefore, it is recommended to optimize the patch thickness, a conclusion that has been supported in previous studies [9,52,53]. Furthermore, it is observed that the difference in the SIF between a single and double reinforcing patch stabilizes when the thickness exceeds 0.006 mm. This observation is reinforced in Figure 9, which depicts the variation in the ratio R with the patch thickness.

(25) $$R=\frac{K_{\infty }^{s}}{K_{\infty }^{d}},$$

where $K_{\infty }^{s}$ and $K_{\infty }^{d}$ are the single- and double-sided repair values of the SIF; thus, it can be observed that as the thickness grows, the ratio R rises asymptotically. The expression implies that the Rose formula can be changed for the double-sided symmetric patch.

(26) $$K_{\infty }^{d}={\;\mathrm{CK}}_{\infty }^{s}$$

The variable C is a function that typically depends on the properties of the host plate, the adhesive bond, and the reinforcing patch. Belhouari et al. [9] formulated this factor analytically, and it can be inferred that C is independent of patch thickness at certain values, specifically, 0.006 mm under plane stress conditions.

In the present study, the increase in patch thickness was achieved by using a double-sided patch. The proportional gain of using a double-sided patch compared with a single-sided patch was defined as the difference in thickness percentage between the two scenarios when the SIF values were the same. The gain values were found to be constant at 50% for various fracture lengths due to the low thickness values and plane stress conditions considered in this study. However, Belhouari et al. [9] noted that this may not hold for plane strain conditions.

The results indicate that the total gain achieved by using a double patch can significantly exceed 50% under both plane stress and plane strain scenarios when considering the gain from eliminating the bending effect in addition to the gain from using a double patch. It is worth noting that for high values of patch thickness, the difference in transferred stresses between a single reinforcing patch and a double reinforcing patch is less significant than for low values of patch thickness.

#### 5.2.3. Effect of Applied Load

**Figure 10:**
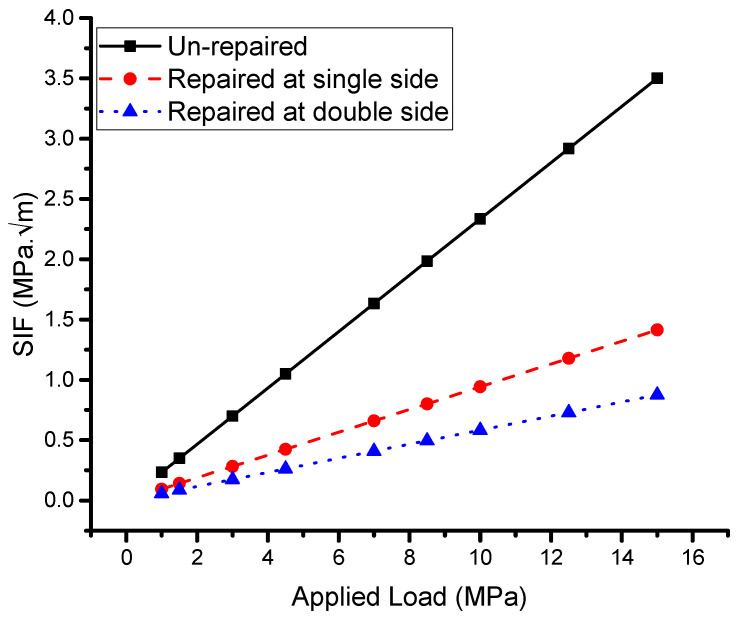
Effect of the applied load.

The results of the analytical modeling calculations of the stress intensity factor (SIF) concerning the applied load are shown in Figure 10. The use of reinforcing patches in repaired specimens led to a reduction in the SIF, which is dependent on the size of the patches and the type of bonded adhesive. In this study, Araldite 2015 was used as the adhesive. The analytical modeling analysis revealed that both the single- and double-sided reinforcing patches were effective in reducing the SIF at the crack’s tip, resulting in an increased lifespan of the plates with the restored patch. However, the reduction in the SIF was affected by the size of the patch and the type of adhesive used. In some cases, adverse effects such as debonding and bending of the repaired plate may occur if a single-sided patch is used. Other reinforcement techniques, such as double-bonded strips or applying compression pre-stresses along the fracture surfaces, can provide additional reductions in the SIF [11].

It is worth noting that the phenomena observed in Figure 7 and Figure 10 are similar, with the main difference being the value of the *x*-axis. In Figure 10, it can be observed that the SIF is approximately constant when the applied load is less than 1 MPa. As the load increases, the SIF also increases, and this increase is more significant in the unrepaired plate. This can be mitigated by using a bonded reinforcing patch, and the single-sided patch was found to be more effective, while the double-sided patch increased the reduction in the SIF by up to 25% to 50%, depending on the crack length and applied load. In Figure 10, it can be concluded that an increase in the applied load leads to an increase in the SIF in all three conditions of the study, and a reduction in the SIF can be achieved by using a repairing patch.

### 5.3. Optimization Results

The analysis utilized L_8_ orthogonal arrays to obtain linear regression equations for the SIF response values, considering the response values of the repair model. ANOVA was performed to assess the statistical significance of each factor’s main effects on the SIF performance, and an optimization study was conducted to identify the optimal quality of each parameter. Contour plots were generated to examine the influences of parameter interactions. Table 3 presents the SIF response values for each parameter combination. The goal of this investigation was to track how SIF values change during the repair of a damaged structure based on the selected parameters. Analytical modeling and analysis were utilized to determine the SIF variation for each factor and level.

#### 5.3.1. Development of Linear Regression Equation 

Using MINITAB 18 and Design Expert 13 software, an arithmetic model based on linear regression equations was identified utilizing the Taguchi OA. A linear polynomial model (regression equations) for the SIF was created based on each factor and is shown in the equations below:

Single-sided

(27) $$\mathrm{SIF}\;\left(\mathrm{MPa}\;\sqrt{m}\;\right)=0.173+0.0059\times \mathrm{Crack}\;\mathrm{Length}-0.178\times \;\;\mathrm{Patch}\;\mathrm{Thickness}+0.03432\times \mathrm{Applied}\;\mathrm{Load},$$

Double-sided

(28) $$\mathrm{SIF}\;\left(\mathrm{MPa}\;\sqrt{m}\;\right)=-0.023+0.0059\times \mathrm{Crack}\;\mathrm{Length}-0.178\times \;\;\mathrm{Patch}\;\mathrm{Thickness}+0.03432\times \mathrm{Applied}\;\mathrm{Load},$$

The percentage deviation for response SIF values in the test cases of single- and double-sided reinforcing patches was determined using linear regression equations (Equations (27) and (28)). The residual errors for each test are presented in Table 4, with the highest errors ranging within ±1.55. The data in Table 4 illustrate the fluctuations in the SIF values of the mathematical model and optimization fits for each run. It is important to note that bonding errors in the measurement method and limited deviation in the analytical modeling in the current work may cause fluctuations in the rate of change in the SIF. 

#### 5.3.2. Analysis of Taguchi Design

##### Main Effect Plot

To evaluate the quality of the repair, the response variable chosen was the SIF of the repaired plate. The aim was to reduce the SIF by optimizing the patch size and adhesive bonding characteristics while keeping them within a specific range. 

**Figure 11:**
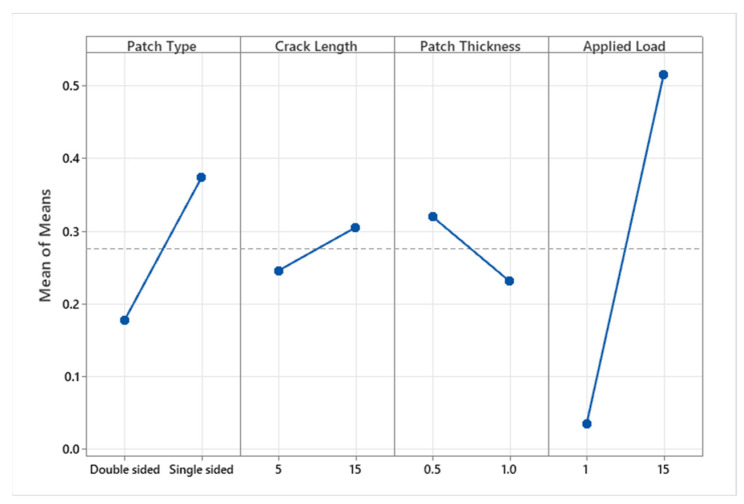
Main effect plot for SIF.

To determine the major effect of each controlled process parameter on the SIF, a main effect plot was created using the MINITAB 18 software. Figure 11 shows the characteristics of the influence of each parameter on the SIF of the repaired plate. When the output level of each parameter is close to the mean line (the horizontal center line), the setting has no discernible effect. On the other hand, the line that has the greatest inclination toward this will be greatly affected. In Figure 11, the load has the most significant effect, followed by the patch type, with a moderate impact. However, the patch thickness has a marginal effect compared with the crack length for the bonded composite material. It is also noteworthy that the crack length is a parameter of the host structure and does not influence the repair performance, whereas the other selected parameters are of the bonded material and significantly impact the SIF reduction. Based on the main effect plot and the selected parameters, the highest SIF reduction is observed at 1 MPa for a double-sided reinforcing patch with a patch thickness of 1 mm. Bonding the aluminum material with the reinforcing patch transfers the shear load and reduces stress concentration, resulting in a reduction in the SIF.

##### Contour Plots

**Figure 12:**
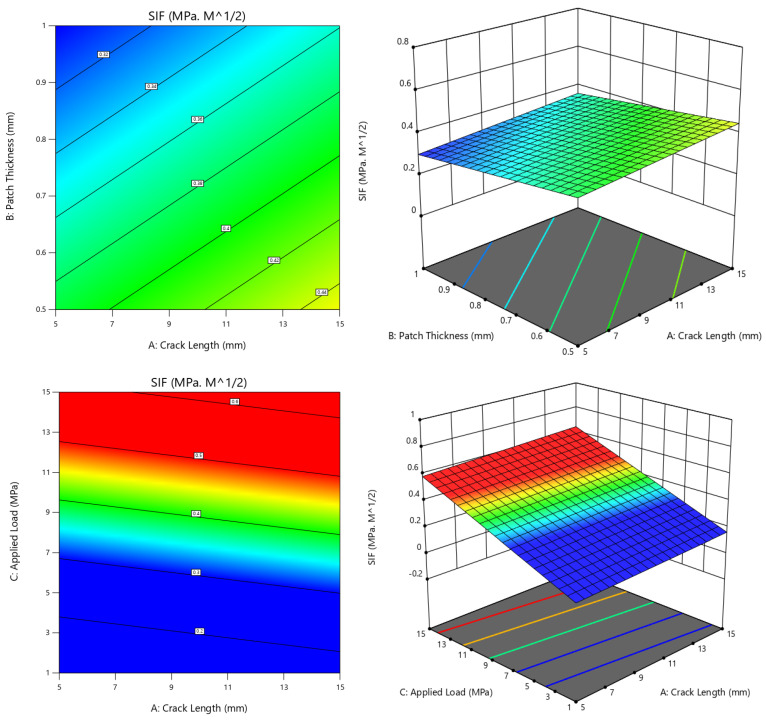

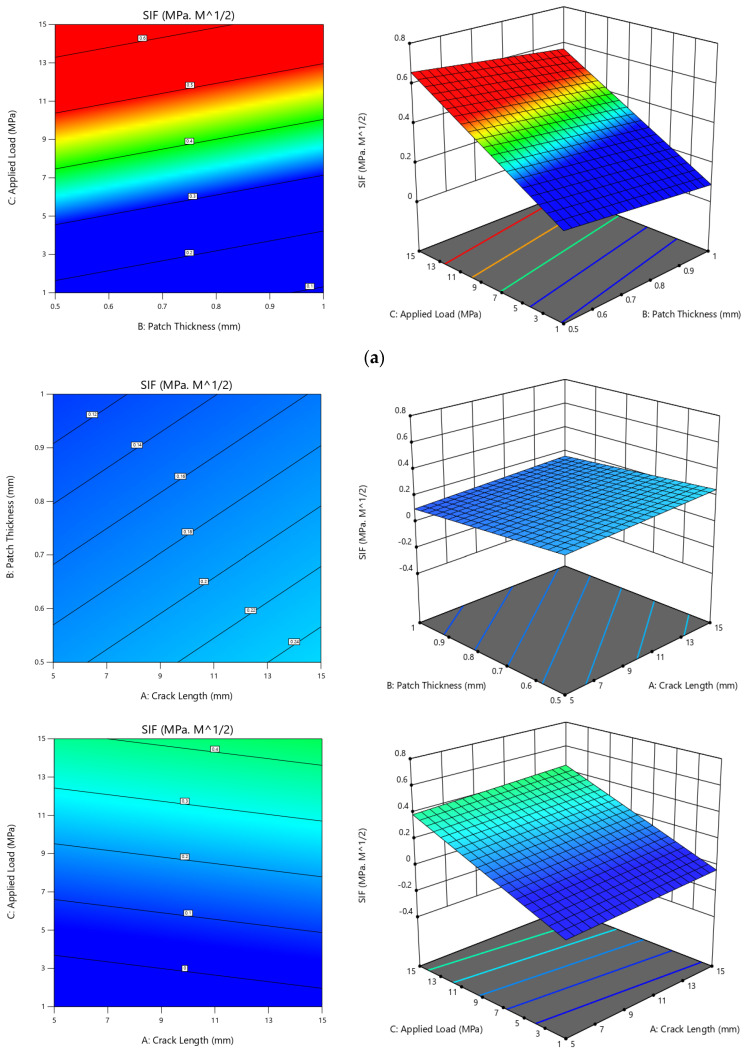

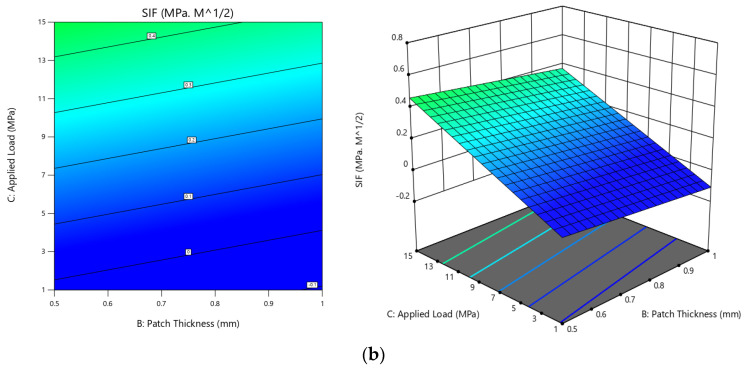
Contours for selected combinations. (**a**) Single-sided; (**b**) double-sided.

Contour plots were generated to show how the SIF value changes for different parameter combinations. The plots were based on the selected parameters and their available combinations. By observing the color variation from dark blue to red in a contour, we can identify the lowest SIF value. Figure 12 presents the contour plots for both single- and double-sided reinforcing patches, with suitable *x*- and *y*-axes. In Figure 12a, three contours (two-dimensional and three-dimensional view) with different axes are shown for the case of a single-sided reinforcing patch. Since crack length is a parameter of the host structure, we chose the *x*-axis value to represent the crack length and used patch thickness and applied load as the *y*-axis. It was found that as the crack length increases, the SIF value increases both before and after the plate is repaired [54]. This was demonstrated in the present case, where the highest reduction in the SIF (≤0.32) was achieved for a crack length of 5 mm and patch thickness of 1 mm, and the applied load was in a smaller range. The combination of patch thickness and applied load showed the maximum reduction for a patch thickness of 1 mm and an applied load of 1 MPa. 

For double-sided reinforcing patches, the contours varied numerically but were similar to the single-sided contours, indicating that this parametric combination would have a greater impact on the variation in the SIF if the plate were repaired with a reinforcing patch (Figure 12b). Additionally, these contour plots show that when there is no applied stress on the cracked plate, the SIF is almost zero, and when it is supposed to bond to a single-sided patch, the SIF is then found to be 0.1, which can be assumed to be almost zero, while for a double-sided patch, the SIF reaches −0.1, which means it has a shear loading effect on the crack’s tip from the reinforcing patches.

#### 5.3.3. Analysis of Variance

Analysis of variance (ANOVA) is a statistical tool that was used to examine the effects of factors and their relationships with the comparison of mean squares in contrast with SIF errors at predefined confidence levels. It helps identify how each element affects the overall variance of the outcomes. Table 5 shows the outcomes of the ANOVA table’s test case for the response factor. A 10% level of significance was used in the analysis, corresponding to a 90% level of confidence. The fourth column of the ANOVA table shows the percentage contribution of each factor variable to the overall variation. This table can be used to analyze each factor’s contribution to each response factor and the influence that greatly impacts the SIF.

ANOVA was used to examine the impacts of bonded parameters. Table 5 shows that the ANOVA analysis displays the SIF reduction for four distinct parameters. Additionally, it shows each factor’s percentage contribution to the overall variation, indicating how much of an impact it had on the outcome. The percentage of variation that each aspect contributes overall shows the amount of influence each component has on the results. The host structure was subjected to bonding under an applied load and other parameters of the bonded patch. The ANOVA results in Table 5 show that the applied load has the highest contribution (72.21%) to the reduction in the SIF. The host structures are more influenced when the load is added. If the plate is unrepaired and the applied load is added to the cracked plate, it will result in crack damage propagation and failure of the whole structure. Therefore, these studies express the impact of applied load on a repaired plate, and it still has a significant impact on the host structure’s breakage.

Regarding the other parameters, the patch type has an impact on the reduction in the SIF, with a contribution of 12.07%. It was found that bonding the patch to both sides of the host structure results in a high impact on SIF reduction. The reinforcing patch thickness also has a significant impact on reducing crack damage propagation and controlling the SIF, with a contribution of 2.46%. Lastly, the host structure’s crack length has the lowest contribution in the present work, with a value of 1.10%, showing no significant impact on reducing the value of the SIF. The error accompanying the ANOVA table is 12.16%.

By using this method, the analytical modeling of the absolute values is given as means and variance variation. This clearly shows that the applied load has a significant impact on SIF reduction. Therefore, it is determined that the Taguchi design results, and analytical modeling are accurate.

#### 5.3.4. Prediction Results 

##### Prediction Plot 

To evaluate the accuracy of the analytical modeling and optimization, the response variables were calculated using regression Equations (27) and (28), and the percentage deviation from the predicted values was determined. The maximum error and significant residual errors for the SIF were found to be 2.55%. Additionally, the R-squared method was used to validate the results of each experiment. This involved several procedures to obtain actual measurements of the variables that were both modeled and predicted. The effects were squared, the real values were subtracted, and the expected values were calculated. The total variance was determined by subtracting the average real value from each actual value, squaring the result, and adding the differences. The R-squared method can be used to compare the following expressions:

(29) $$R^{2}=1-\frac{{\mathrm{SS}}_{\mathrm{RES}}}{{\mathrm{SS}}_{\mathrm{TOT}}}=1-\sum_{i}\frac{{\left(y_{i}-{\hat{y}}_{i}\right)}^{2}}{{\left(y_{i}-{\bar{y}}_{i}\right)}^{2}}$$

Equation (29) reveals that the R-square can be obtained by subtracting the sum of squared errors from one and then dividing the sum of squared errors by the total sum of squares. In this study, the R-square values for the SIF were found to be 87.84%, indicating that the mathematical model developed using the selected parameters has a strong impact on the damaged structural repair. 

**Figure 13:**
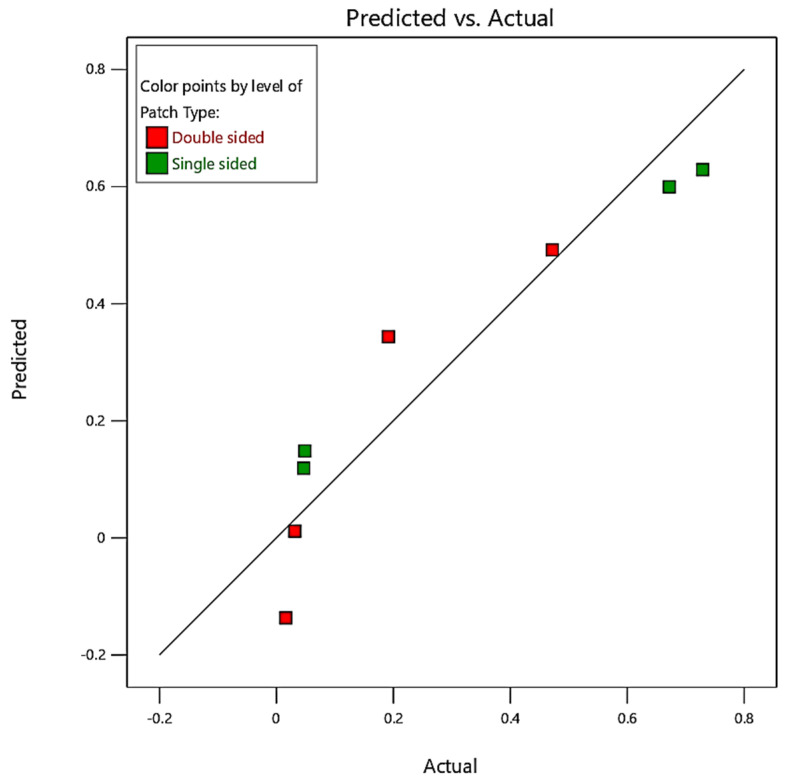
Prediction of SIF results through the Taguchi design.

Figure 13 illustrates the prediction plot of SIF values (the dots) through the Taguchi design. The prediction plot of SIF values is important in structural analysis as it provides insights into the behavior of cracks under various parameters. The SIF is a measure of the stress concentration at the tip of a crack and is influenced by factors such as the patch type, crack length, patch thickness, and applied load. In a prediction plot, these variables are typically plotted against the SIF, which is usually represented on the *y*-axis. The plot shows how the SIF varies with changes in the variables used. For example, the plot may show that the SIF increases as the crack length increases or as the applied load increases. These trends and patterns observed in the plot can provide insights into the behavior of cracks and help in the design and analysis of structures. For instance, the plot can be used to determine the critical crack size and the maximum allowable load. It can also be used to evaluate the effectiveness of different repair methods by comparing the predicted SIF before and after the repair. Finally, it can be used to make informed decisions in the design and analysis of structures and to evaluate the effectiveness of repair methods. 

##### Optimal Parameters Prediction

**Figure 14:**
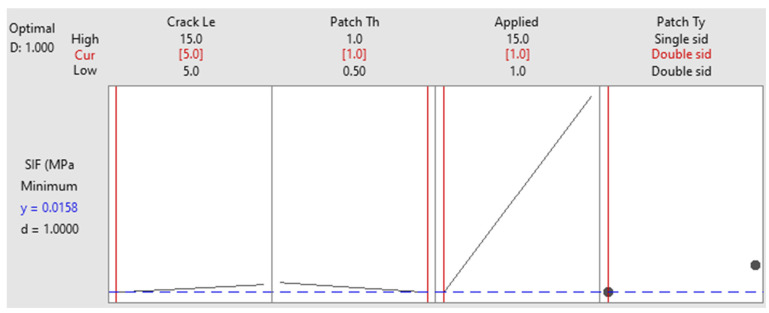
Response optimization with fitted parameters.

The response optimization study was conducted to obtain the best possible results and outcomes from the selected parameters and levels of this study. Based on the response optimization analysis, it recommended the best possible combination of parameters and their levels to achieve the optimum value of the SIF, which can be seen in Figure 14. As a result of the optimization study, the lowest SIF value of 0.0158 MPa·m^1/2^ was achieved, which is a practical and safe value. The results were obtained by combining the following parameters: a double-sided reinforcing patch, crack length of 5 mm, patch thickness of 1 mm, and applied load of 1 MPa. Indeed, it is well known that both sides of a patch will result in a higher stress intensity reduction at the crack’s tip due to perfect bonding. Regarding the other parameters, the patch thickness is shown as 1 mm, which is equal to the thickness of the host structures. However, an increase in the thickness of the patch will result in an increment in the total weight, which was discussed in the previous sections. The applied 15 MPa is a maximum load, and this load has a larger impact on the cracked plate due to thin structures; hence, 1 MPa is recommended for the optimum solution. Lastly, the crack length optimized value is 5 mm due to the ratio of the healthy structure being higher and the impact on the stress intensity on the crack’s tip being smaller while applying the load. The validation tests were performed by comparing the results of the analytical modeling, which showed that the SIF value is 0.0162 MPa·m^1/2^. The red line and the blue dashed line of each combination and interaction from these two lines will predict the optimum value of parameter from there limits. These parametric changes are like the ideal value attained using the Taguchi design approach. 

## 6. Conclusions

In this study, we investigated the efficacy of a bonded reinforcing patch in crack healing using analytical modeling. Our analysis revealed that using a double patch leads to a significant reduction in the SIF compared with a single patch. The relative difference between the SIF values for the double and single patches remains nearly constant, and as patch thickness increases, the SIF drops asymptotically, while the R ratio rises. Once a certain value is reached, the R ratio no longer depends on the thickness, and optimizing the patch thickness is recommended. With an increasing crack length and decreasing patch thickness, the advantage of thickness gain increases. At the asymptotic SIF value, this advantage can exceed 50% for short lengths. Furthermore, we observed that the effect of the applied load on the SIF increases, while the reduction in the SIF decreases.

In addition, we successfully optimized the repair of an edge-cracked plate using the Taguchi design method with a Taguchi OA. We utilized the SIF obtained through analytical modeling and the parameters of the double-sided reinforcing patch, crack length, patch thickness, and applied load for optimization. The optimized parameters produced the minimum SIF, indicating the best-fitted parameters. Our study found that the maximum thickness of the single- and double-sided reinforcing patches had varying effects on achieving the optimum (reduced) value of the SIF.

## Figures and Tables

**Table 1:** Factors and their levels.

Parameter	Patch Type	Crack Length (mm)	Patch Thickness (mm)	Applied Load (MPa)
Level 1	Single-sided	5	0.5	1
Level 2	Double-sided	15	1	15

**Table 2:** Taguchi orthogonal array.

Run		1	2	3	4	5	6	7	8
Coded Values	A	1	1	1	1	2	2	2	2
	B	1	1	2	2	1	1	2	2
	C	1	1	2	2	2	2	1	1
	D	1	2	1	2	1	2	1	2

**Table 3:** Taguchi orthogonal array with analytical results.

Run	Patch Type	Crack Length (m)	Patch Thickness (m)	Applied Load (MPa)	SIF (MPa. m^1/2^)
1	Single-sided	5	0.5	1	0.048594527
2	Single-sided	5	0.5	15	0.728977448
3	Single-sided	15	1	1	0.046735684
4	Single-sided	15	1	15	0.672010353
5	Double-sided	5	1	1	0.015778775
6	Double-sided	5	1	15	0.191442929
7	Double-sided	15	0.5	1	0.031467357
8	Double-sided	15	0.5	15	0.472010353

**Table 4:** Fits and diagnostics for L_8_ observations.

Run	Selected Parameter				$\mathrm{SIF}(\mathrm{MPa}\sqrt{m})$	Fit	SE Fit	Resid.	Std. Resid.
	Patch Type	Crack Length (m)	Patch Thickness (m)	Applied Load (MPa)					
1	Single-sided	5	0.5	1	0.049	0.149	0.127	−0.100	−1.01
2	Single-sided	5	0.5	15	0.729	0.629	0.127	0.100	1.01
3	Single-sided	15	1	1	0.047	0.119	0.127	−0.072	−0.73
4	Single-sided	15	1	15	0.672	0.600	0.127	0.072	0.73
5	Double-sided	5	1	1	0.016	−0.137	0.127	0.152	1.55
6	Double-sided	5	1	15	0.191	0.344	0.127	−0.152	−1.55
7	Double-sided	15	0.5	1	0.031	0.012	0.127	0.020	0.20
8	Double-sided	15	0.5	15	0.472	0.492	0.127	−0.020	−0.20

**Table 5:** Analysis of variance.

Source	DF	Seq SS	Contribution	Adj. SS	Adj. MS	F-Value	*p*-Value
Patch type	1	0.077150	12.07%	0.077150	0.077150	2.98	0.183
Crack length	1	0.007047	1.10%	0.007047	0.007047	0.27	0.638
Patch thickness	1	0.015760	2.46%	0.015760	0.015760	0.61	0.492
Applied load	1	0.461696	72.21%	0.461696	0.461696	17.82	0.024
Error	3	0.077717	12.16%	0.077717	0.025906		
Total	7	0.639369	100.00%				

## Data Availability

Not applicable.

## Abbreviations

2D	Two-dimensional
3D	Three-dimensional
ANOVA	Analysis of variance
ERR	Energy relies rate
FE	Finite element
LEFM	Linear elastic factor mechanics
MS	Mean square
NSIF	Normalized stress intensity factor
SCF	Stress concentration factor
SE	Standard error
SERR	Strain energy release rate
SIF	Stress intensity factor
SS	Sum of square
VIF	Variance inflation factor
